# Contextual drivers of HIV risk among young African women

**DOI:** 10.1002/jia2.25302

**Published:** 2019-07-22

**Authors:** Sanyu A Mojola, Joyce Wamoyi

**Affiliations:** ^1^ Department of Sociology and Woodrow Wilson School of Public and International Affairs Princeton University Princeton NJ USA; ^2^ Department of Sexual and Reproductive Health National Medical Research Institute Mwanza Tanzania

**Keywords:** HIV prevention, adolescent girls, young women, Africa, hyper‐endemics, interventions

## Abstract

**Introduction:**

Significant progress has been made in the African HIV pandemic; however, the pace of incidence decline has slowed or stalled in many East and Southern African countries, especially among young women. This stall is worrying because many countries have burgeoning youth populations. There is an important window of opportunity to halt the epidemic as well as the potential for millions more infections if primary prevention efforts are not strengthened.

**Discussion:**

Many hyper‐endemic settings have been exposed to numerous interventions; however, HIV incidence among young women has remained high. In this paper, we characterize the intervention context and examine how it can be strategically utilized to maximize HIV prevention interventions among young women. We begin by examining how contextual dynamics drive HIV risk. We illustrate how epidemiological contexts, gendered normative and economic contexts, and environmental contexts work synergistically to make young women especially vulnerable to HIV infection. We then examine how these contexts can undermine HIV prevention interventions. Finally, we discuss the importance of fully mapping out the intervention context to enhance the effectiveness of HIV prevention interventions.

**Conclusions:**

Understanding an intervention context, and how its features work together to amplify young women's risk in hyper‐endemic settings can contribute to sustained momentum in reducing HIV incidence among young women and help to limit the reach of the HIV pandemic into new generations of Africans.

## 1 Introduction

Despite significant progress over the last few decades, sub‐Saharan Africa continues to bear the brunt of the HIV/AIDS pandemic, with two‐thirds of the 1.8 million new HIV infections, and 70% (an estimated 660,000 deaths) of AIDS related mortality 1. Adolescent girls and young women are disproportionately affected; an estimated 7000 are newly infected each week and 75% of new infections among 15‐ to 19‐year olds are in girls 1. In South Africa alone, there were an estimated 113,000 new infections among women aged 15 to 24 2. Furthermore, many high prevalence countries in East and Southern Africa have between a third to almost half of their populations under the age of 15 3, and there is a slow down or stall in the pace of decline in new infections 4. Stalling HIV epidemics combined with burgeoning youth populations present both a challenge and an important window of opportunity where the epidemic can be halted or yield to dramatic increases of those in need of life‐long medication 5, 6.

Recent years have brought a growing recognition of the spatial concentration of hyper‐endemics (settings with persistently high HIV incidence, and/or HIV prevalence exceeding 15% of the adult population 7, 8), and the significance of social context to focus HIV prevention interventions and improve HIV incidence control 9, 10, 11, 12, 13, 14, 15. However, despite numerous interventions 16, 17, 18, 19, 20, incidence remains high. How might intervention outcomes be improved? In this paper, we build on previous research by characterizing the intervention context, and examining how it can be strategically utilized to maximize HIV prevention interventions among young women. We begin by describing how contextual dynamics drive HIV hyper‐endemics, we then illustrate how they can undermine prevention interventions, and conclude by discussing how fully mapping an intervention context can contribute to more effective HIV prevention interventions among young women. While our conceptual framework is applicable to other settings, we draw on African examples to illustrate our points.

## 2 Discussion

### 2.1 How social contexts drive HIV hyper‐endemics

**Figure 1:**
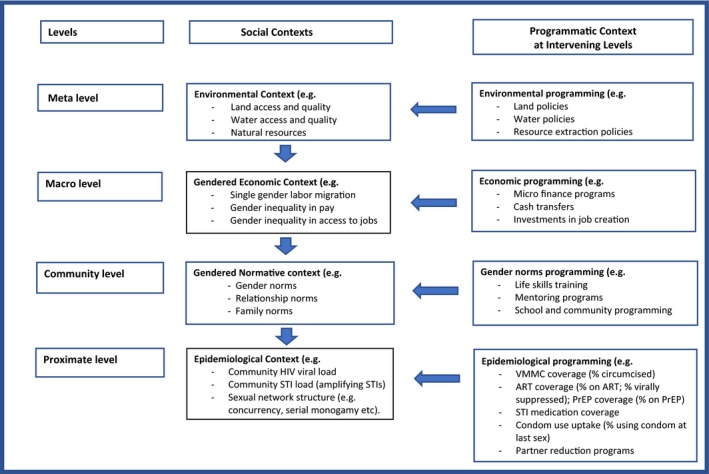
**Intervention context**

Many hyper‐endemic settings share similar social contexts that interact synergistically to create a dangerous HIV risk environment for girls transitioning to adulthood. Figure 1 illustrates the different contexts which we discuss in turn.

#### 2.1.1 The epidemiological context

Hyper‐endemics are often characterized by interacting epidemiological factors that work together to make even a single sexual encounter risky for young women 21. For example in Kenya, 65% of new infections are concentrated in nine of its 47 counties 22. Young people (15 to 24) account for half (52%) of new infections in the country's highest incidence and prevalence counties which are Homa Bay (26% prevalence), Siaya (25% prevalence) and Kisumu (20% prevalence) 23, located in Nyanza province. Nyanza also has an amplifying sexually transmitted infection (STI) epidemic; 57% and 38% of women and men respectively have herpes simplex virus type 2 (HSV‐2) 24. STIs such as HSV‐2 are associated with a significant increase in HIV acquisition risk 25, 26, 27. Furthermore, the sexual network structure in Nyanza creates vulnerability with high levels of concurrency 28, 29. Despite a risky epidemiological context, uptake of programming targeted at this level (epidemiological programming) is low. In Homa Bay county, 44% of men were uncircumcised, and almost half of those with multiple partners reported not using a condom at last sex. HIV testing among key populations such female sex workers and men who have sex with men was low. While most (63%) adults living with HIV were on antiretroviral therapy (ART), viral suppression was 55% 23. KwaZulu‐Natal (KZN), South Africa has similar combinations of interacting epidemiological factors and the HIV prevalence is 24%. About 51% of 25‐ to 29‐year‐old women, and 44% of 30‐ to 34‐year‐old men are living with HIV 30, 31. Like Nyanza, KZN also has an amplifying STI epidemic (syphilis, gonorrhoea, chlamydia, *Trichomonas vaginalis*). STI prevalence is 13%, and young women under age 25 are at significantly higher risk 32. About 77% of men have not been circumcised 33, and while viral suppression among those on medication was high among teenagers (68%) and 20‐ to 24‐year olds (87%), overall, ART use was less than 45% among those aged 15 to 34 34.

In sum, in hyper‐endemic settings like these, young women's sexual lives unfold in epidemiological contexts with high community viral loads of HIV and amplifying STIs. Furthermore, high prevalence means they are likely to encounter a partner with HIV, and their most likely sexual partners – men in their 20s and 30s – generally have low rates of circumcision, report relatively low rates of condom use, and among those living with HIV, have relatively low viral suppression. This combination of interacting epidemiological factors places young women at significant risk for HIV acquisition 21, 35.

#### 2.1.2 The gendered normative context

When layered on to a dangerous epidemiological context, the gendered normative context works to further amplify young women's HIV risk. Social norms regulate sexual behaviour by setting “group level expectations for appropriate behavior that result in negative sanctions for people who violate them.” [36, p. 1283, 13]. Gendered community norms set expectations around women's autonomy, ideal or accepted sexual network structures (e.g. concurrency or serial monogamy), gender power and material exchanges within relationships 37, 38, 39, 40, 41, 42. Community members can be sanctioned through stigma and shaming (e.g. of women seen to have too many relationships or men who do not provide for their partners), leaving relationships, and gender‐based violence 43, 44, 45, 46. Further, young women are not operating as individuals, but rather, are embedded within families/households and communities through which gendered norms and expectations may be primarily exerted 47, 48.

Hyper‐endemic HIV risk environments share similar gendered normative contexts characterized by high levels of women's autonomy in entering and exiting relationships, but unequal gender power norms within them that are exacerbated when they are transactional 37, 38, 39, 40. Transactional sexual relationships are common in many hyper‐endemic settings 5, 37, 38, 39, 41. A survey in Kisumu, Nyanza's capital found that 72% of men gave almost 10% of their monthly income to girlfriends in the form of cash, meals, drinks, gifts, transportation and rent support 49 suggesting that men's provision was a normative expectation. Men who can provide are often older, and age‐disparate relationships are associated with HIV acquisition among young women due to limited relationship agency resulting in limited leverage to use or insist on prevention technologies such as condoms and HIV testing 5, 37, 50, 51, 52.

It is important to note that gender and relationship norms also amplify men's HIV risk. Masculinity norms in many historically polygamous cultures are supportive of concurrency, and may also lead to men's lower uptake of HIV testing and treatment 5, 53, 54, 55, 56. In Uganda, for example, concurrent men found couple HIV testing challenging 55. Gendered norms are also embedded in community institutions such as health facilities which are often women‐focused and sometimes neglect men 57, 58, 59.

Overall, the gendered normative context encourages young women to pursue the riskiest partners in their community – men in their 20s and 30s who are able to provide, but who are also more likely to afford and have cultural support in seeking multiple partners, and who are less likely to be reached by epidemiological programming.

#### 2.1.3 Gendered economic contexts and environmental contexts

Many hyper‐endemic settings also have similar economic configurations characterized by widespread inequality and poor employment opportunities 31, 60, 61. This contributes to high circular male labour migration to cities, mines, farms, on the road and on water for more lucrative work; women, meanwhile, have relatively limited employment and wage income 5, 62, 63, 64, 65, 66, 67. Labour migration has been linked to high rates of concurrency, with patterns of reunion and separation providing regular opportunities for HIV transmission, and serving as bridges between sexual networks in different locations 62, 63, 65, 66, 67, 68, 69. Female sex work is often symbiotic with labour migration, and places many women at particularly high risk 66, 70. Young women with migrant partners such as fishermen or truck drivers are also especially vulnerable 65, 66, 67. Gendered economies thus amplify the HIV risk environment for young women by creating economic circumstances that can increase their likelihood of being involved in transactional and/or concurrent partnerships with high risk men, as well as commercial sex relationships. This results in a sexual network structure that further exacerbates young women's vulnerability.

Finally, it is important to note that the environmental context is often an underlying driver of gendered economic contexts. For example, unsustainable agricultural livelihoods may contribute to male out‐migration; widows displaced from land may turn to sex work; new resource extraction may attract many more men to an area; and an imbalanced lake eco‐system may exacerbate fishermen's migratory patterns leading to extended sexual networks and increased vulnerability for women 5, 65, 70, 71.

While many features of these social contexts are not unique to hyper‐endemic settings, they are distinguished by how they synergistically work together to significantly amplify young women's HIV vulnerability.

### 2.2 How social contexts undermine HIV prevention interventions

There is now a widespread consensus that strategic combinations of multi‐level HIV prevention approaches are the way forward, and many hyper endemic settings have been exposed to numerous interventions over the past few decades 13, 14, 72, 73, 74, 75, 76, 77, 78, 79. The most successful of these have been the widespread roll‐out of ART which has led to large‐scale reductions in HIV viral load and AIDS mortality, along with voluntary medical male circumcision (VMMC) 31, 80, 81, 82, 83, 84. In the following section we discuss how contexts can undermine otherwise efficacious interventions.

#### 2.2.1 How epidemiological contexts undermine interventions

Epidemiological contexts are often the Achilles heel of HIV prevention interventions. Major pre‐exposure prophylaxis (PrEP) trials among young women in high incidence settings have had limited success 18, 19, 20. This might be in part because other interacting factors operating in the epidemiological context were not concurrently engaged through epidemiological programming such as STI test and treat, VMMC, condom use campaigns, and male partner outreach for HIV testing and treatment. Epidemiological programming contributes to reducing the community STI and HIV viral load, thus creating an enabling environment 16 for PrEP to work. Singularly focusing on PrEP places undue weight on the intervention and young women's high adherence to it to protect themselves from HIV. This “single bullet” approach makes intervention success even more challenging when considering the amplifying effect of gendered normative contexts.

#### 2.2.2 How gendered normative contexts undermine interventions

Many HIV prevention interventions do not adequately work to meaningfully alter the gendered normative context that will ultimately determine prevention uptake and its long‐term sustainability 5, 20, 85, 86. Unequal gender power norms, reinforced by age‐disparate relationships limit young women's leverage and willingness to regularly use and negotiate prevention technologies such as condoms or PrEP 5, 37, 43, 63, 85, 86, 87. This is exacerbated in long‐term relationships where the potential for repeated exposure to HIV exists alongside love and trust. Indeed this might explain why couple PrEP interventions have been effective 88, 89, 90. Intervention success may be continually undermined when it is not paired with programming to create enabling community norms governing prevention technologies in ways that engage both women and men. Relevant community institutions such as families and schools which may only support abstinence, and health facilities which might limit access for young unmarried women are also important to engage.

#### 2.2.3 How gendered economic and environmental contexts undermine interventions

Many promising micro‐finance or cash transfer interventions have been conducted with limited impact on HIV incidence with few exceptions 91, 92, 93, 94, 95, 96. Economic interventions can be undermined by synergies between the gendered normative and gendered economic context. Intervention design implicitly or explicitly substitutes male partner provision for intervention programme provision. This aligns with young women's limited economic opportunities and their limited ability to purchase desired goods for themselves. While interventions substitute material provision, however, they do not substitute what that provision also expresses – love and commitment 5, 63, 97, 98, 99. This is likely exacerbated in hyper‐endemic settings with large male migrant populations, where migrants express commitment through remittances or material provision. Furthermore, the short‐term nature of interventions suggests that when they end, without associated efforts to stabilize young women's income, risk may be heightened with the renewed search for a partner. Finally, environmental interventions to improve land use or water quality, or to increase employment through opening up new resource extraction economies can undermine HIV prevention interventions if they serve to reinforce gendered economies which predominantly employ and differentially compensate men.

### 2.3 Mapping and strategically utilizing the Intervention context

As Figure 1 illustrates, new interventions enter into a large ecology of pre‐existing social and programmatic contexts that may enable, undermine or have a neutral effect on their ability to achieve their goals. The synergistic nature of contextual drivers of hyper‐endemics highlights the importance of analysing and utilizing the intervention context to achieve incidence control.

An important first step is to map out the pre‐existing intervention context in a given setting, and then locate the new intervention – and its intended mechanisms to reduce HIV incidence – within it. This will enable intervention designers to clearly see potential barriers and/or catalysts to their proposed intervention. Fully mapping the intervention context also enables designers to assess how much weight is being placed on the new intervention to achieve incidence control, and whether a longer duration or multi‐level approach might increase the chances of success 13. Finally, mapping the intervention context prior to intervention initiation would enable more systematic post‐intervention analyses of why similar interventions worked in some settings and not in others 13, 15, and ultimately, guide decisions about whether and under what conditions an intervention should be scaled up.

Mapping the intervention context also enables a strategic utilization of pre‐existing features of social and programmatic contexts to increase the chances of intervention success. For example, intervening at multiple levels may be beyond the funding scope of an intervention; however, as noted earlier, numerous interventions are often ongoing in hyper‐endemic settings 100. When mapped, programming synergies become visible; they may preclude the need for a combination approach within a given intervention, or enable strategic planning of the most effective combinations given what already exists. Combination prevention interventions such as DREAMS would require different kinds of coordination with pre‐existing programming than those aimed at one level (e.g. VMMC or PrEP), but which might need new supportive programming at different levels. An important coordinating role could be played by governments and sub‐regional local authorities who typically engage in multi‐sectoral planning, as many interventions within an intervention context aimed at HIV prevention might align well with broader community development goals.

## 3 Conclusions

Each year, millions of adolescent African girls begin their sexual debut in hyper‐endemic settings where one‐fifth to one‐third will be HIV positive by the time they are in their late 20s and early 30s. This commentary has examined how contextual drivers might contribute to stalling epidemics, and how they might be deployed to maximize HIV prevention. Understanding how contexts synergistically work together in hyper‐endemic settings, and fully mapping and strategically utilizing the intervention context could enable sustained momentum in reducing HIV incidence among young women, and limiting the reach of the HIV pandemic into new generations of Africans.

## Competing interests

The authors report no competing interests.

## Authors’ contributions

S.M. wrote the first draft of the manuscript. J.W. contributed to writing the manuscript. Both authors approved the final draft.
